# Simulation Study on the Impact of Melt Track Overlap Rate on the Forming Result During the Selective Laser Melting of Ti-6Al-4V Alloy

**DOI:** 10.3390/ma18102314

**Published:** 2025-05-15

**Authors:** Chen Liu, Weidong Huang, Hui Wang, Zebin Lin, Zhiyuan Lai

**Affiliations:** 1 Fujian Key Laboratory of Intelligent Machining Technology and Equipment, Fujian University of Technology, Fuzhou 350118, China; 2230104017@smail.fjut.edu.cn (C.L.); hwd@fjut.edu.cn (W.H.); 2210104001@smail.fjut.edu.cn (Z.L.); 2230104003@smail.fjut.edu.cn (Z.L.); 2 School of Mechanical and Automotive Engineering, Fujian University of Technology, Fuzhou 350118, China

**Keywords:** selective laser melting, Ti-6Al-4V alloy, numerical simulation, melt track overlap rate, process optimization

## Abstract

The melt track overlap rate is a crucial process parameter in selective laser melting (SLM). It exerts a substantial influence on the forming quality of Ti-6Al-4V alloy. This paper investigates the impact of different melt track overlap rates on the quality of Ti-6Al-4V samples formed by SLM through a combination of simulation and experimentation. The findings reveal that if the overlap rate is overly small (<14.3%), the powder at the bottom of the overlap zone cannot be fully melted, thereby forming pores. Additionally, the densification effect causes the actual powder spreading thickness to be greater than the theoretical one. Consequently, when the overlap rate is less than 20%, it is challenging to ensure a close bond between the upper and lower melt tracks. Both the simulation and experimental results demonstrate that an excessive melt track overlap rate will lead to grain growth in the overlap zone and reduce the tensile properties of the sample. Thus, both excessive and insufficient overlap rates are detrimental to the density and mechanical properties of the formed sample. The multi-layer and multi-track model established in this study effectively replicates the actual processing conditions. The retention of the temperature field of the first layer makes the simulation of the melt tracks of the second layer more realistic and reliable. This provides a reference basis for accurately predicting the forming quality of SLM using numerical models in the future.

## 1. Introduction

Selective laser melting (SLM), an additive manufacturing technology with great prospects, employs a high-power-density laser beam as the heat source [1,2]. It can fully melt metal powder along the preset trajectory in a short time [3,4]. Through successive layering, a three-dimensional metal part with high density and superior mechanical properties can be rapidly fabricated [5,6]. In comparison with traditional processing methods, SLM can manufacture metal components with complex structures more conveniently. It also has the advantages of less material wastage, lower environmental pollution, and higher processing flexibility, conforming to the concept of modern green processing [7,8,9]. Ti-6Al-4V alloy is characterized by high specific strength, good low-temperature performance, and excellent corrosion resistance. It is widely used in various fields, such as aerospace, biomedical, and automobile manufacturing [10,11,12]. Nevertheless, titanium alloys are prone to react with oxygen in the air during processing, making it difficult for titanium alloy parts produced by traditional processing methods to achieve outstanding mechanical properties [13]. Undoubtedly, SLM is a preferable method for processing complex-shaped Ti-6Al-4V alloys, which has led many scholars to conduct research on this technology [14,15].

In the SLM processing process, changes in process parameters such as laser power, laser scanning speed, scanning distance, and powder layer thickness will directly affect the quality of SLM formed parts [16,17,18]. The SLM forming cost of titanium alloy materials is relatively high. Optimizing process parameters through simulation first is beneficial for ensuring the quality of formed parts and reducing the scrap rate [19,20]. At present, some teams at home and abroad have carried out experimental and simulation studies on the SLM forming process [21,22]. The team of Yu-lung Lo [23] conducted a double-track simulation study on the SLM processing process of 316L stainless steel through COMSOL software. They discussed the influence of the scanning distance and scanning length of two melt tracks on the peak temperature and morphology of adjacent melt tracks. Finally, it was found that the scanning length of melt tracks has little influence on the peak temperature of adjacent melt tracks, while the scanning spacing of melt tracks will seriously affect the peak temperature of melt tracks. It was also pointed out that the overlap rate between adjacent melt tracks should be 25% of the width of the molten track as the best. Wang et al. [24] studied the pore segregation phenomenon generated at the overlap of adjacent melt tracks through experiments and simulations. It was found that the porosity generated at the boundary of the remelted zone of adjacent melt tracks is about eight times that inside the melt track. Moreover, the number of segregated pores will significantly decrease as the spacing between melt tracks increases. He et al. [25] used software to model the SLM forming process. Considering many influencing factors such as fluid flow, heat transfer, melting, solidification, and surface tension, they proposed a scanning strategy that can prevent excessive energy leakage of the Gaussian heat source on the moving interface, effectively improving the melting efficiency of metal powder during processing. Ge et al. [7] used Flow-3D software to simulate and discuss the internal situation of a single-track molten pool of Ti-6Al-4V alloy under different powers, and analyzed the defects in the melt track. It was proposed that the threshold of linear laser energy density in the processing of Ti-6Al-4V alloy should be around 200 J/mm. The team of Elham Mirkoohi [26] modeled through finite element software, and revealed the influence of different types of laser heat sources on the melting degree of the powder bed. It was shown that when the linear laser energy density is too high (>0.3 J/mm) or too low (<0.1 J/mm), balling will occur, but the mechanisms for forming balling are different.

In most of the existing literature, the research on the track overlap rate of SLM melt tracks is mainly based on single-layer simulation models, and there are relatively few studies on multi-layer simulations [27,28]. During the SLM forming process, the molten pool in the second layer scanning is influenced by the residual temperature field in the first layer, leading to an increase in the width and depth of the molten pool [29]. Thus, to obtain the true simulation result of the molten pool in the second layer, it is necessary to accurately preserve the residual temperature and its gradient in the first layer, which is rather challenging [30]. Currently, some models increase the preheating temperature of the substrate by 100 K to simulate the residual temperature after the first layer scanning [31,32]. However, this is different from the actual processing situation and cannot well reproduce the real state of the molten pool and melt track in the second layer during processing. Therefore, how to establish a real and reliable multi-layer model to accurately simulate the SLM forming process is a research direction worthy of exploration [33].

This study will take the melt track overlap rate of adjacent melt tracks in the SLM forming process of Ti-6Al-4V alloy as the research object and conduct experimental research and simulation analysis. This paper discusses the influence of different melt track overlap rates on the density and mechanical properties of formed parts through experiments. Then, using the Fluent 2023 R1 software, a multi-layer and multi-track simulation model of SLM was established. Compared with traditional models, this model effectively retains the residual temperature of the first-layer melt track through data interaction among multiple software programs. As a result, the melt track morphology and the melt track temperature field presented in the simulation results of the second-layer melt track are closer to the actual processing conditions. This further improves the accuracy of the numerical model for SLM multi-layer simulation. Under the premise of ensuring the accuracy of the simulation model, this model was used to analyze the formation reasons of internal pores in the overlap zone and the temperature change in the overlap zone at different overlap rates. This study provides analytical support for the influence of the track overlap rate during the SLM forming process on the forming quality of Ti-6Al-4V samples. It also offers a reference basis for the subsequent promotion of using simulation models to select an appropriate track overlap rate.

## 2. Experimental Conditions and Methods

### 2.1. Materials and Equipment

**Figure 1:**
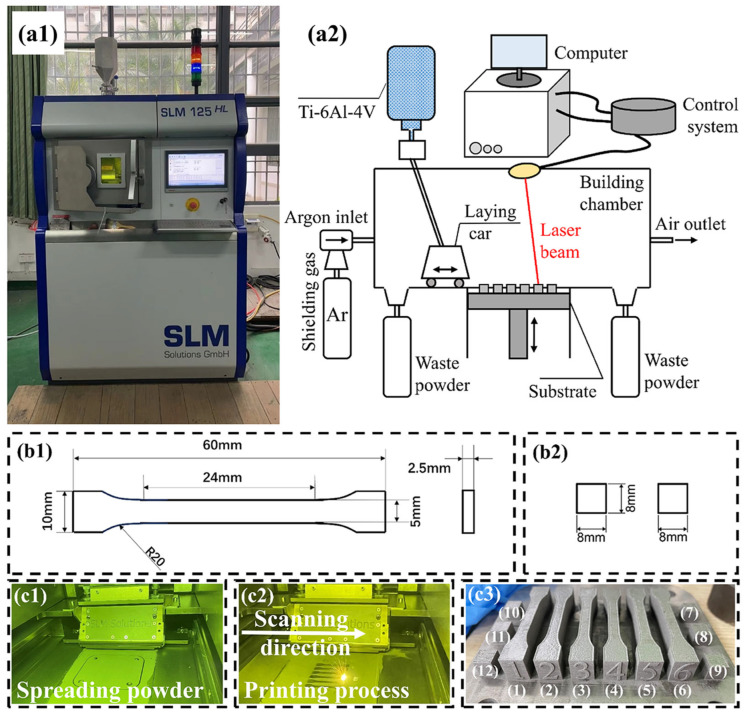
Experimental equipment and process. (**a**) Equipment and principle: (**a1**) experimental equipment, and (**a2**) forming principle of equipment. (**b**) Sample dimensions: (**b1**) dimension of the tensile sample, and (**b2**) dimension of the small square. (**c**) Forming process and outcome: (**c1**) powder spreading outcome, (**c2**) forming process, and (**c3**) final formed sample.

The percentages of the weight fractions of various elements in the Ti-6Al-4V alloy powder used in the experiments of this paper are shown in Table 1. The powder diameters of the Ti-6Al-4V used in the experiment range from 10 to 30 µm [34]. The selective laser melting device SLM 125 (SLM Solutions, Lübeck, Germany) is utilized to fabricate experimental samples. The experimental equipment and forming principle are depicted in Figures 1(a1) and 1(a2), respectively.

### 2.2. Experimental Method

To ensure accuracy, the experiments were carried out using argon as a protective gas with purity over 99.9%. The substrate employed in the SLM experiment was a titanium substrate and the preheating temperature of the substrate was 200 °C [35]. The process parameters are set by Materialise Magics 22.03 software as shown in Table 2. Since this study did not involve changes in these parameters, the parameters selected in Table 2 are the best process parameters provided by the SLM 125 manufacturer. In this experiment, the melt track overlap rate was calculated by the width of a single melt track and different scanning distances. The formula for the melt track overlap rate is:

(1) $$Overlap\;rate\left(\%\right)=\left(1-\frac{Scanning\;distance}{Weld\;width}\right)\times 100\%$$

A total of six different scanning distances were designed, as shown in Table 3. At different laser scanning distances, a total of six small square samples and six tensile samples were formed. The laser power and scanning speed used for forming each sample are the same. The dimensions of the tensile samples and small square samples are shown in Figures 1(b1) and 1(b2), respectively. Figure 1(c1,c2) show the SLM forming process of the samples, and the final shape of the samples is shown in Figure 1(c3). The forming quality of the surface melt tracks of the formed samples No. 7–12 was observed by optical microscope (OM) and scanning electron microscope (SEM), and the density of the samples was measured by the Archimedes method. The principle of the Archimedes method is to first measure the mass of the sample part using a load cell, and then place the sample part in water to measure the volume of water displaced by the sample part. The density of the sample part is obtained by dividing the former by the latter. An Instron 8801 (Dongri Instrument, Taiwan, China) testing machine was used to analyze the strength of tensile samples Nos. 1–6 at a tensile rate of 0.5 mm/min. Four tensile specimens can be obtained from each sample part, which means that each sample part has undergone four tensile tests. Finally, electron backscatter diffraction (EBSD) was used to characterize and analyze the microscopic grain structure of the melt tracks at different overlap rates.

## 3. Numerical Model

The fluid is regarded as an incompressible Newtonian fluid (dρ/dt = 0) to simplify the mass conservation equation. Since the flow velocity of the molten liquid is not high (v < 100 m/s), the model ignores the heat dissipation generated by viscous dissipation to improve the overall computational efficiency.

### 3.1. Numerical Implementation

**Figure 2:**
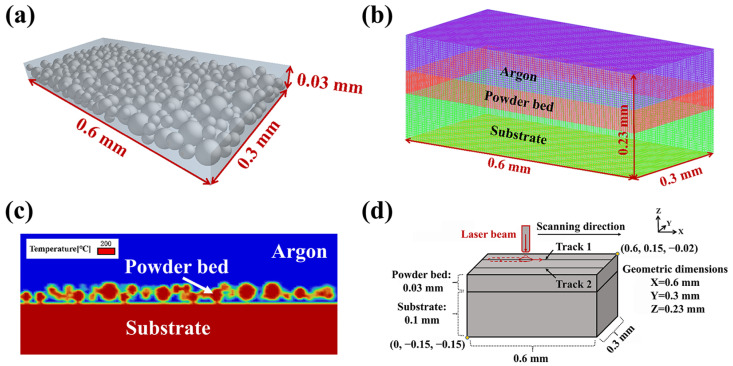
Numerical model: (**a**) powder bed model, (**b**) division and dimensions of the computational domain, (**c**) substrate preheating to 200 °C and argon gas filling, and (**d**) coordinate system setting for the simulation process.

In this simulation, the discrete element method was used to construct a powder bed model. The Ti-6Al-4V metal powder was defined as particles with diameters distributed between 10 and 30 µm. Some thermal physical parameters of the metal powder particles are shown in Table 4. Subsequently, a powder bed model with a size of 0.6 mm long × 0.3 mm wide × 0.03 mm thick was generated by EDEM 2022.2 software. The powder bed model is shown in Figure 2a. The ICEM-CFD 2023 R1 software was used to define the calculation domain size as 0.6 mm long × 0.3 mm wide × 0.23 mm thick, and the meshes in the powder area and 0.02 mm above it were encrypted [36]. The argon gas was 0.13 mm thick (including the calculation domain where the powder is located, that is, the Argon gas wrapped the powder), and the substrate part was 0.1 mm thick. Figure 2b shows the divided calculation domain, where the total number of elements was 517,275 and the total number of nodes was 537,776. During the simulation process, the powder and substrate were preheated to 200 °C before laser irradiation, as shown in Figure 2c. Argon gas with a concentration of more than 99.9% was filled above the powder and into the powder. The traveling path and speed of the laser light source were controlled by a motion function [37]. The spacing between adjacent laser beams was changed by changing the y-coordinate value of the center of the laser light source, thereby controlling the overlap rate of adjacent molten tracks. The schematic diagram of the simulation process coordinates is shown in Figure 2d. During the simulation process, the length of each molten track was controlled at 0.4 mm, and there was no printing time interval between two molten tracks in the same layer. The final simulation results and contour plots are presented after being processed by CFD-Post 2023 R1.

### 3.2. Discrete Particle Contact Model

The discrete element method (DEM) was employed to construct a simulation model of the powder bed. Suppose that Ti-6Al-4V particles were hard spheres. The interaction force between two spheres would be of an impulsive nature, and particles would exchange energy using collisions. In this case, the movement of a single particle can be categorized into two types: rotation and translation. The governing equations for these two motions can be expressed, respectively, as [39]:

(2) $$m_{i}\frac{dv_{i}}{dt}=F_{T},\;I_{i}\frac{d\omega_{i}}{dt}=T_{i}$$

where *m_i_* represents the mass of powder particle *i*, **v***_i_* denotes the migration velocity of powder particle *i*, *I_i_* is the moment of inertia of particle *i*, **ω***_i_* indicates the angular velocity of powder particle *i*, **F***_T_* and **T***_i_* are, respectively, the total force and total torque acting on particle *i*. Additionally, there exists energy dissipation in the energy transfer between particles, mainly caused by rolling friction. In the subsequent modeling using EDEM 2022.2 software, the employed model is the standard friction model (neglecting the partial slip contained in friction). Here, the rolling friction primarily depends on the rotational speed when particles are in contact. Therefore, the torque **τ***_i_* in the rolling friction model can be expressed as [39]:

(3) $$\tau_{i}=-\mu_{r}F_{n}R^{*}\omega_{rel}$$

where *µ_r_* represents the rolling friction coefficient, **F***_n_* denotes the normal force at the contact point, R* indicates the radius of the contacting particles, and **ω***_rel_* is the rotational angular velocity. Based on the standard contact model, the force **F***_t_* in the shear direction between particles is [40]:

(4) $$F_{t}=-\frac{2}{3}\left(8G\sqrt{R^{*}\delta_{n}}\right)\delta_{t}$$

where *G* is the rigidity modulus of the powder, and **δ***_n_* and **δ***_t_* are the sums of the forces on the particles in the normal direction and the shear direction, respectively. Additionally, to account for the influence of van der Waals forces between powder particles, the Johnson–Kendall–Roberts (JKR) model is introduced. This model describes the influence of the dryness and humidity of powder on the dissipation of acting force. The dissipative force **F***_JKR_* can be expressed as [40]:

(5) $$F_{JKR}=-4\sqrt{\pi \gamma E^{*}}a^{\frac{3}{2}}+\frac{4E^{*}}{3R^{*}}a^{3}$$

where *γ* is the surface energy, E* is the equivalent Young’s modulus, and **a** is the particle acceleration.

### 3.3. Macroscopic Heat and Mass Transfer Model

#### 3.3.1. Model of Laser Heat Source

**Figure 3:**
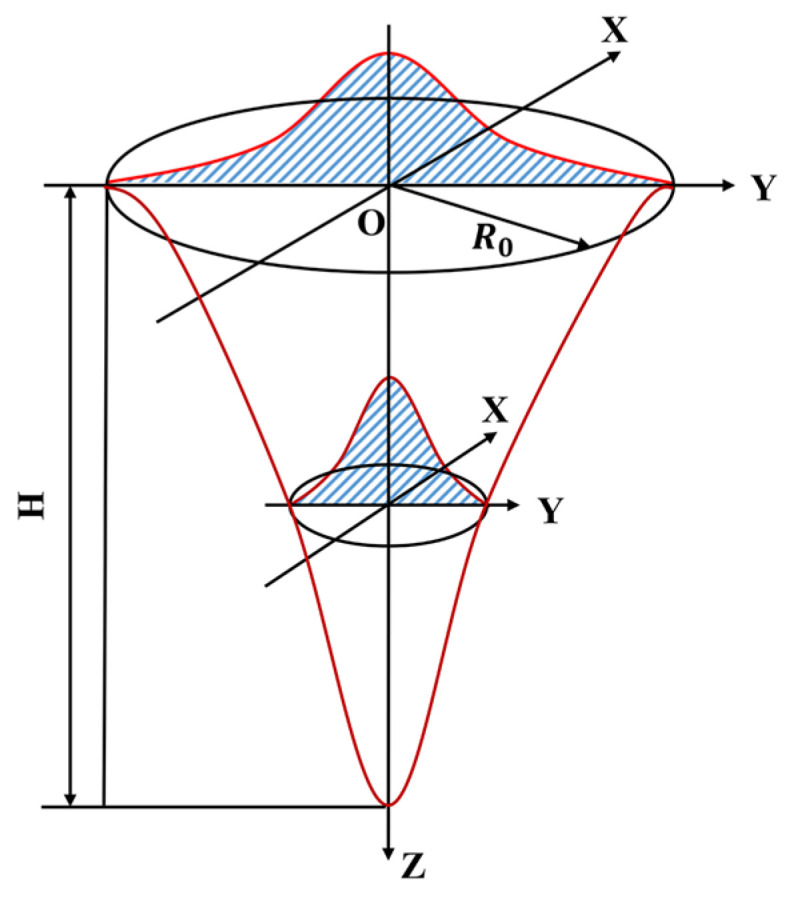
Geometric schematic diagram of the Gaussian rotating body heat source model.

The SLM processing process involves a relatively complex energy transfer process. In this, the energy density distribution of laser irradiation on the powder bed surface can be represented by a planar Gaussian heat source model. The equation is expressed as follows [41]:

(6) $$f\left(x,y\right)=\frac{1}{2\pi \sigma_{x}\sigma_{y}}exp-\left[\frac{{\left(x-u_{x}\right)}^{2}}{2\sigma_{x}^{2}}+\frac{{\left(y-u_{y}\right)}^{2}}{2\sigma_{y}^{2}}\right]$$

where *σ_x_* = *σ_y_*. *σ_x_* and *σ_y_*, respectively, correspond to the widths in the x-direction and y-direction of the heat source distribution. In the equation, *u_x_* and *u_y_* are the coordinate positions of the center of the heat source. In the equation, x and y are the coordinate positions of any point of the heat source. In the SLM forming process, the distribution of the laser heat source is not only on the powder surface layer but also penetrates into the powder bed. Therefore, a Gaussian volume heat source needs to be introduced. In the direction perpendicular to the scanning direction, the energy density of the light source varies greatly. The cross-sectional area of the heat source will gradually decrease as the heat transfer depth increases, and the heat source energy density of each cross-section also presents a Gaussian distribution, which conforms to the characteristics of the Gaussian rotating body heat source model. Figure 3 is a geometric schematic diagram of the Gaussian rotating body heat source model.

This heat source model can well describe the heating state inside the powder bed and can reflect a more realistic morphology of the molten pool. Its mathematical expression is [41]:

(7) $$q\left(x,y,z\right)=\frac{9Q}{\pi HR_{0}^{2}\left(1-\frac{1}{e^{3}}\right)}exp-\frac{9}{R_{0}^{2}\log\left(\frac{H}{z}\right)}\left(x^{2}+y^{2}\right)$$

where *Q* represents the total power of the heat source, *H* is the height of the heat source, and *R*_0_ is the opening radius of the heat source.

#### 3.3.2. Governing Equations

(1) Heat transfer equation

In the SLM process, considering the principle of energy conservation and the entropy increase, the Boltzmann space heat transfer evolution equation is introduced [42]:

(8) $$\frac{\partial T}{\partial t}=\alpha \nabla^{2}T$$

where *T* is the temperature field, *t* is the time, *α* is the thermal diffusivity, and ∇^2^ is the Laplacian operator.

(2) Momentum equation

The rate of change in fluid momentum concerning time is equal to the sum of all forces acting on the fluid. The law of conservation of momentum is expressed as follows [42]:

(9) $$\frac{\partial \left(\rho V\right)}{\partial t}+\nabla \cdot \left(\rho VV\right)=-\nabla P+\nabla \cdot \left(\tau \right)+\rho g+F$$

where *ρ* is the density of the molten metal, **V** is the velocity of the fluid flow in the molten pool, **P** is the static pressure on the infinitesimal element, *τ* is the viscous stress tensor generated by the molecular viscosity acting on the surface of the infinitesimal element, and **g** and **F** are the gravitational and body forces acting on the infinitesimal element, respectively. For the molten metal, the viscous stress is proportional to the deformation rate of the fluid, which is expressed as follows [42]:

(10) $$\tau =\mu \left[\left(\nabla V+\nabla V^{T}\right)-\frac{2}{3}\nabla \cdot VI\right]$$

where *μ* is the viscosity and **I** is the unit matrix.

(3) Energy equation

In the SLM process, energy must also obey the law of conservation of energy, expressed as follows [43]:

(11) $$\frac{\partial \left(\rho C_{p}T\right)}{\partial t}+\nabla \cdot \left(\rho VC_{p}T\right)=-\frac{\partial \rho ∆H}{\partial t}-\nabla \cdot \left(\rho V∆H\right)+\nabla \left(k\nabla T\right)+S_{H}$$

where *C_p_* is the specific heat capacity at constant pressure of the material, Δ*H* is the latent heat of phase change of the material, *k* is the thermal conductivity coefficient of the material, and **S***_H_* is the momentum source term.

#### 3.3.3. Surface Tension and Recoil Pressure

The surface tension of the molten metal *γ* is related to the temperature field. The relationship between them can be expressed as [44]:

(12) $$\gamma =\gamma_{l}+\frac{d\gamma }{dT}\left(T-T_{l}\right)$$

where *γ_l_* is the magnitude of the surface tension at temperature *T_l_*. According to the Clausius–Clapeyron equation, the recoil pressure **F***_r_* can be obtained as [44]:

(13) $$F_{r}\left(T\right)=0.54P_{0}\exp\left(L_{v}M\frac{T-T_{b}}{RTT_{b}}\right)$$

where **P**_0_ is atmospheric pressure, *L_v_* is the latent heat of vaporization, *M* is atomic mass, *T_b_* is boiling point temperature, and *R* is the universal gas constant.

#### 3.3.4. Marangoni Effect

The Marangoni effect arises because, under the action of surface tension, there will be a shear force flowing from the high-temperature region to the low-temperature region inside the molten metal liquid. This shear force **F***_m_* can be expressed as [44]:

(14) $$F_{m}=2V\frac{d\gamma }{dT}\left[\nabla T-n\left(n\nabla T\right)\right]$$

where *V* is the volume fraction of Ti-6Al-4V alloy, and **n** is the interface normal vector between the material and the shielding gas.

#### 3.3.5. The Definition of “Mushy Zone”

**Figure 4:**
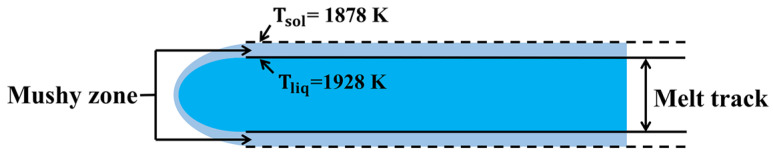
Schematic diagram of the location of the ”mushy zone”.

In the overlap zone between two adjacent melt tracks, the metal powder will experience two fluctuations in the temperature field. When the overlap zone is large, the metal powder in the overlap zone will be completely melted. However, when the overlap zone is small or there is no overlap, the metal powder in the overlap zone cannot be fully melted. When the overlap zone is small, the temperature of the metal powder in this zone is often between the solidus line and the liquidus line. Assume that this zone is the “mushy zone”, as shown in Figure 4.

The degree of melting of the mushy zone is defined by a liquid fraction *β*, which can be calculated by linear interpolation as follows [45]:

(15) $$\left\{\begin{array}{c} 0,\;T\leq T_{sol}\\ \beta =\frac{T-T_{sol}}{T_{liq}-T_{sol}},T_{sol}<T<T_{liq}\;\\ 1,T\geq T_{liq} \end{array}\right.$$

In the formula, *T_sol_* is the solidus temperature, and *T_liq_* is the liquidus temperature. The change in latent heat in this region is [45]:

(16) $$∆H=\beta \cdot L_{m}$$

In the formula, ∆*H* is the enthalpy change of latent heat, and *L_m_* is the latent heat of fusion of metal. This will affect the energy source term in the energy equation. The momentum source phase **S***_M_* in the mushy zone between solid and liquid phase changes can be expressed as [45]:

(17) $$S_{M}=-\frac{{\left(1-\beta \right)}^{2}}{\left(\beta^{3}+\delta \right)}A_{mush}V$$

where *δ* is a very small number to prevent the denominator from being zero. *A_mush_* is the momentum coefficient. Under this model, *A_mush_* is taken as 10^12^ to eliminate the non-physical movement of the fluid in the mushy zone.

#### 3.3.6. Boundary Conditions

The boundaries in the simulation included the boundary of the computational domain and the boundary between the gas and liquid phases. The thermal boundary condition of the computational domain boundary can be expressed as [38]:

(18) $$\lambda \frac{\partial T}{\partial n}=-\varepsilon \sigma \left(T^{4}-T_{0}^{4}\right)-h_{conv}\left(T-T_{0}\right)$$

where *n* is the boundary temperature change function, *ε* is the thermal radiation emissivity, *σ* is the Stefan–Boltzmann constant, *T*_0_ is the ambient temperature, and *h_conv_* is the convection coefficient. The thermal boundary condition of the gas and liquid phases can be expressed as [38]:

(19) $$\lambda \frac{\partial T}{\partial n}=q_{L}-\varepsilon \sigma \left(T^{4}-T_{0}^{4}\right)-h_{conv}\left(T-T_{0}\right)-q_{evap}$$

where *q_L_* is the heat flux density of the laser on the surface, and *q_evap_* is the heat lost due to evaporation. The *q_evap_* can be expressed as [38]:

(20) $$q_{evap}=0.82\frac{∆H^{*}}{\sqrt{2\pi MRT}}P_{0}\exp\left(∆H^{*}\frac{T-T_{LV}}{RTT_{LV}}\right)$$

(21) $$∆H^{*}=∆H_{LV}+\frac{\gamma_{C}\left(\gamma_{C}+1\right)}{2\left(\gamma_{C}+1\right)}RT$$

where *T_LV_* is the vaporization temperature, ΔH* is the enthalpy of vapor flowing at sonic speed, Δ*H_LV_* is the latent heat of evaporation, and *γ_C_* is a proportionality coefficient.

### 3.4. Flowchart of Simulation Process

**Figure 5:**
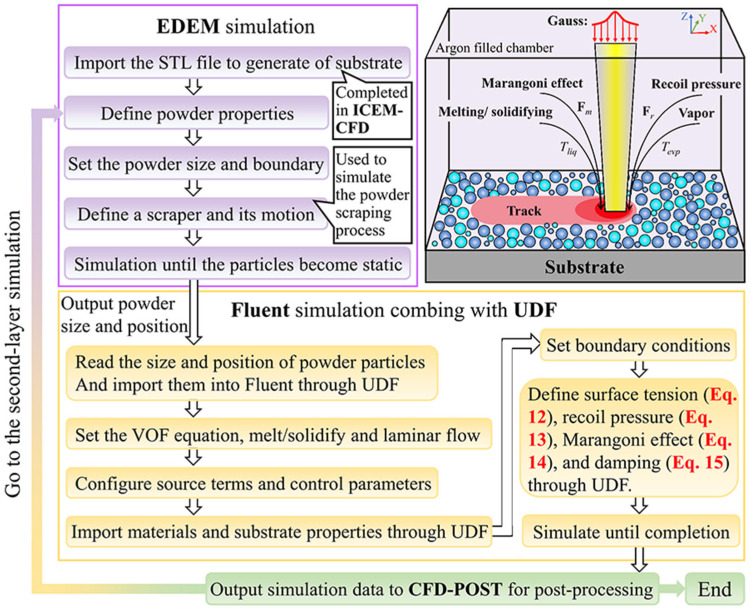
Simulation flowchart.

Since the established model is relatively complex, involving the movement of powder, liquid, and gas as well as two phase changes, a process flow chart of the simulation is presented to show the entire implementation process, as shown in Figure 5.

## 4. Results and Discussion

### 4.1. Investigation of the Forming Quality of Ti-6Al-4V Samples

#### 4.1.1. Surface Morphology and Density of Samples

**Figure 6:**
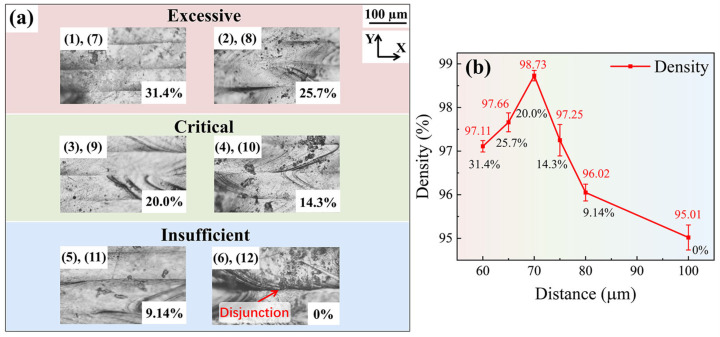
Surface morphology and density of Ti-6Al-4V samples with different overlap rates: (**a**) surface melt track morphology, and (**b**) the test results of the sample density.

The forming quality of the surface melt tracks of the formed samples (XY plane) was observed by an OM. Figure 6a shows the surface conditions of the formed samples at different melt track overlap rates and is arranged in the order of Table 3. As can be seen from the surface melt tracks of sample No. 6, when the melt track overlap rate is 0% at this scanning distance, there is an obvious “gully” between the two melt tracks because there is no overlap between the melt tracks. It can be inferred that the density of the sample obtained at this overlap rate will be the lowest. The surface overlap of other samples is good, and there is an overlapping zone between the two melt tracks. Subsequently, the density of each sample was measured by the Archimedes method. The change trend of the sample density is shown in Figure 6b. As can be seen from the figure, with the increase in the melt track overlap rate, the density of the sample first increases and then decreases. When the melt track overlap rate is 20%, the density of the formed sample is the highest, reaching 98.73%.

It can be seen that both excessive and too-small overlap rates will lead to a decrease in the density of the sample. In previous studies by Wang et al. [23,24], the reason for the decrease in the density of the sample parts caused by an excessively high overlap rate of the melting tracks was attributed to pore segregation. The following research of this study will focus on the influence of the overlap rate between two melt tracks on the porosity of the samples and the welding condition between the upper and lower layers.

**Figure 7:**
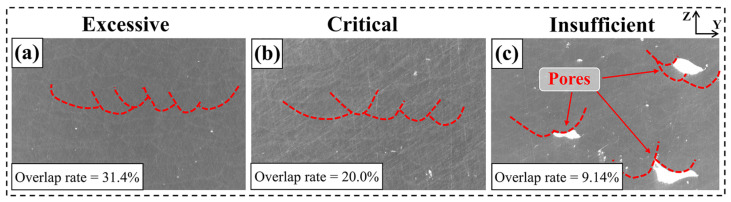
YZ cross-section of Ti-6Al-4V samples with different overlap rates: (**a**) overlap rate = 31.4%, (**b**) overlap rate = 20.0%, (**c**) overlap rate = 9.14%.

Figure 7 shows the OM images of the YZ cross-section of the sample after grinding and polishing. It can be seen that at an excessively melt track overlap rate (overlap rate = 31.4%) and a critical melt track overlap rate (overlap rate = 20.0%), macroscopic pores are almost invisible at the macroscopic scale, as shown in Figure 7a,b. However, at a lower melt track overlap rate, many macroscopic pores appear in the melt track cross-section, as shown in Figure 7c. Through observation, it can be found that these pores are mainly located below the overlap zone of two melt tracks. Thus, it can be inferred that the main source of these pores may be due to insufficient melting depth in the overlap zone, which causes the powder below the overlap zone not to be completely melted and results in pores.

#### 4.1.2. Tensile Strength of Ti-6Al-4V Samples

**Figure 8:**
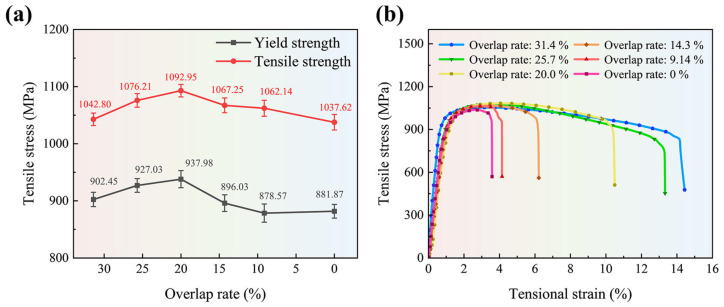
Tensile test results of Ti-6Al-4V samples with different overlap rates: (**a**) tensile strength and yield strength, and (**b**) the curve of stress and strain.

Through tensile tests on tensile samples No. 1 to No. 6, the yield strength and tensile strength of each sample obtained are shown in Figure 8a. Judging from the changing trend of tensile strength, as the melt track overlap rate decreases (from 20% to 0%), the tensile strength shows a gradually decreasing trend (1092.95 ± 11.05 MPa–1037.62 ± 13.65 MPa). This is mainly due to the increase in internal pores leading to stress concentration during tension. In the case of an excessive overlap rate (31.4% and 25.7%), the tensile strength of the sample also shows a decreasing trend (1092.95 ± 11.05 MPa–1042.80 ± 10.20 MPa). The reason is that the excessive overlap zone leads to slow heat dissipation inside the overlap zone and causes excessive grain growth (β-Ti). It is noteworthy that due to the characteristics of rapid melting and rapid solidification of metal powders in SLM processing, a large temperature gradient exists during the forming process. Tensile samples processed under such conditions will retain residual stresses that contract inward from both ends. Since the direction of this residual stress is opposite to the direction of the tensile stress during tensile testing, the tensile samples formed by SLM processing are higher (about 80–100 MPa) in both yield strength and tensile strength in tensile stress tests compared to those obtained by other processing methods. Figure 8b shows the tensile curves of each sample in the experiment. Among them, the sample with an overlap rate of 20% has a tensional strain of 10.41%, and the sample with an overlap rate of 31.4% has a tensional strain of 14.55%. However, the tensile strength of the sample with an overlap rate of 20% (1092.95 ± 11.05 MPa) is about 50 MPa higher than that of the sample with an overlap rate of 31.4% (1042.80 ± 10.20 MPa). This is mainly caused by a higher cooling rate; more fine acicular grain structure α’-Ti will be precipitated in the β columnar grain region. Finer grains have higher yield strength and ultimate strength, but the ductility will be lower than the sample with an overlap rate of 31.4%. From the above comparisons and analyses, it can be seen that if an appropriate melt track overlap rate can be selected, the yield strength and tensile strength of the formed samples can be improved to a certain degree.

**Figure 9:**
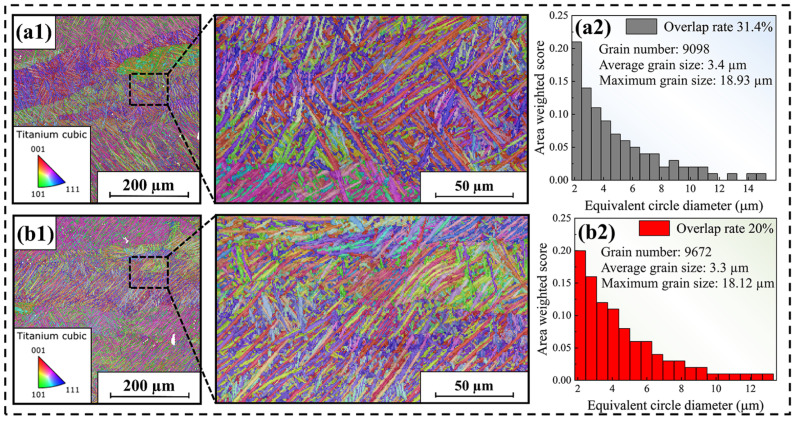
Results of electron backscatter diffraction. (**a**) Sample with an overlap rate of 31.4%: (**a1**) microscopic grain map, and (**a2**) grain size statistics. (**b**) Sample with an overlap rate of 20%: (**b1**) microscopic grain map, and (**b2**) grain size statistics.

EBSD was employed to characterize the microscopic morphology of the surface of the formed sample when the overlap rate was 20% and 31.4%. The imaging area is 500 µm × 500 µm, as depicted in Figure 9(a1,b1). By conducting grain statistics in the entire imaged area, as shown in Figure 9(a2,b2), it can be observed that when the overlap rate is 31.4%, the average grain size (3.4 µm) inside the formed sample is coarser than that when the overlap rate is 20% (3.3 µm). This is because as the overlap rate increases, heat dissipation inside the weld bead becomes difficult. This provides time for the growth of β-Ti columnar crystals, causing them to become coarser. Coarse grains will lead to a reduction in the number of grain boundaries and the corresponding grain boundary energy, resulting in a decline in the strength of the sample, but the corresponding tensile deformation amount will increase to some extent. Consequently, if one wants to increase the strength of the formed sample parts, an overly large overlap rate should not be utilized in SLM processing.

### 4.2. Numerical Model Verification

**Figure 10:**
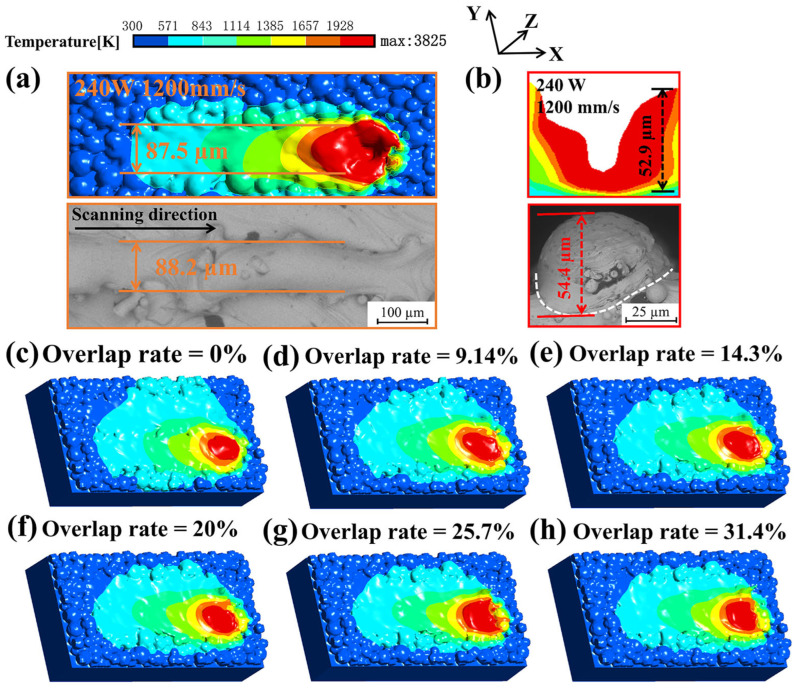
Comparison of single-track simulation results and experimental results. (**a**) Comparison of melt track width. (**b**) Comparison of melt track depth. Double-track simulation results: melt with track overlap rate of (**c**) 0%, (**d**) 9.14%, (**e**) 14.3%, (**f**) 20%, (**g**) 25.7%, and (**h**) 31.4%.

According to the melt track overlap situation that occurs in the experiment, six samples with different laser scanning spacing of 100 µm, 80 µm, 75 µm, 70 µm, 65 µm, and 60 µm are designed, corresponding to adjacent melt track overlap rates of 0%, 9.14%, 14.3%, 20%, 25.7%, and 31.4%, respectively. By comparing the results of the single-track simulation with the experimental results, it can be seen that the melt width of the single-track molten pool in the simulation is 87.5 µm, and the width of the single molten track measured in the experiment is about 88.2 µm, as shown in Figure 10a. Figure 10b compares the depth of the single-track simulation model and the actual molten track. The depth of the molten track in the simulation model is 52.9 µm, and the melt depth of the single track measured in the actual experiment is about 54.4 µm. Figure 10c–h shows the results of the double-track simulation, which are in good agreement with the experimental results shown in Figure 6a. Through the comparison of simulation results and experimental results, it can be seen that there is a high degree of agreement between the two conditions. Therefore, it is feasible to further study the internal pore changes caused by different overlap rates between double tracks through this simulation model.

### 4.3. Effect of Melt Track Overlap Rate on the Porosity of Ti-6Al-4V Samples

#### 4.3.1. Analysis of the Welding State Between Double Melt Tracks

**Figure 11:**
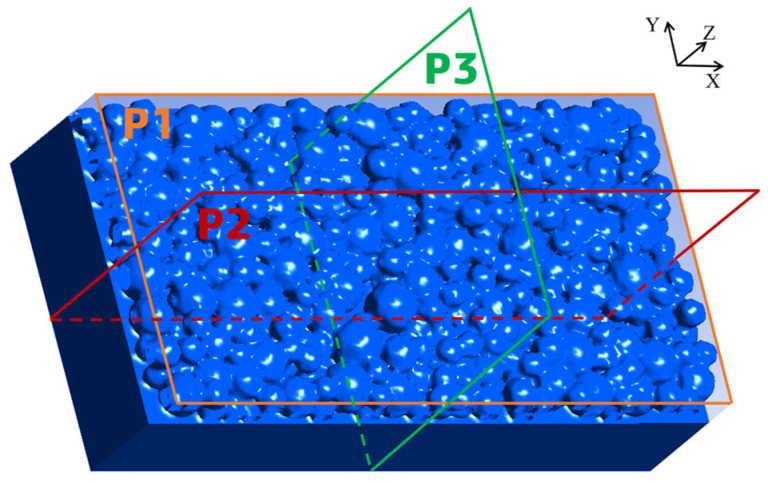
Setting of three observation planes in the simulation results.

**Figure 12:**
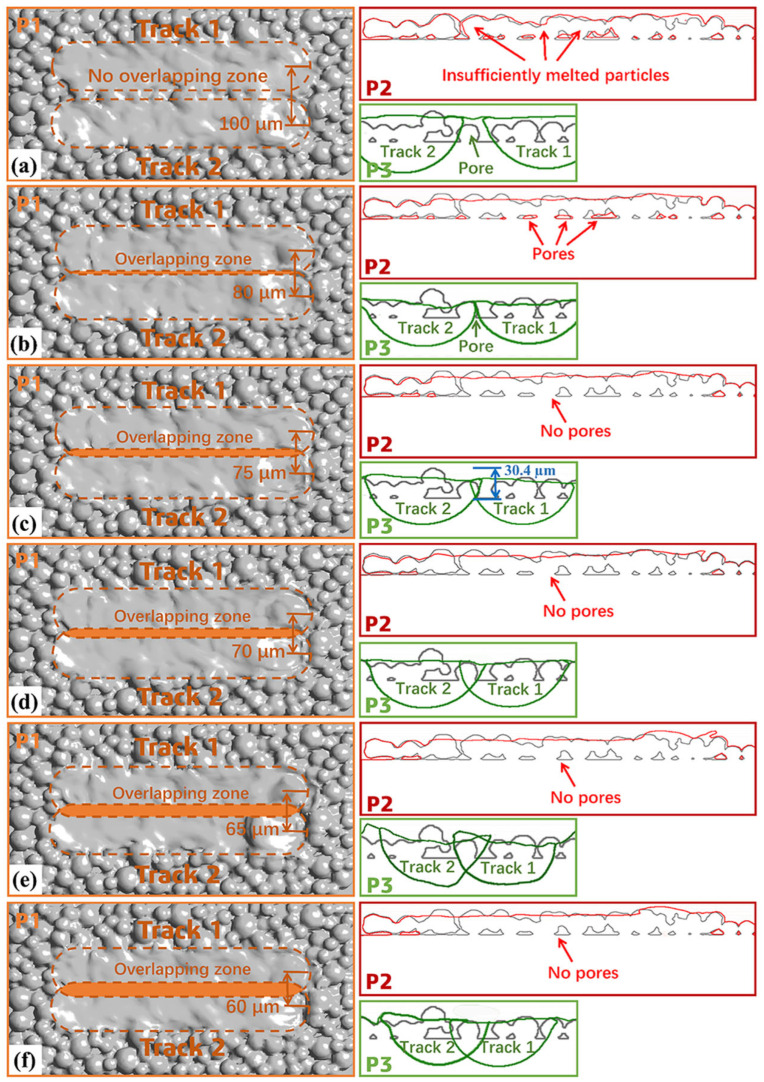
Simulation results of melt tracks with different track overlap rate: (**a**) overlap rate = 0%, (**b**) overlap rate = 9.14%, (**c**) overlap rate = 14.3%, (**d**) overlap rate = 20%, (**e**) overlap rate = 25.7%, and (**f**) overlap rate = 31.4%.

For convenient observation, three mutually perpendicular planar perspectives, P1, P2, and P3, are set in the simulation results, as shown in Figure 11. The important sectional contours in the P2 and P3 views are extracted. The gray contour lines in P2 and P3 represent the contour lines of metal powder particles in the initial state when the laser is not irradiated. The red contour lines represent the powder state after melting, and the green contour lines are the contour of the molten pool. The simulation results are shown in Figure 12.

As can be seen from Figure 12a, when the melt track overlap rate is 0%, there is no effective overlapping zone between melt tracks. From P1, it can be seen that between the two melt tracks, there is an “unmelted zone”, with incompletely melted metal powder particles in between. From the red contour line in the P2 cross-section, it can also be seen that the metal powder particles inside this “unmelted area” almost maintain their original spherical shape. Therefore, there will be a large number of pores inside the part formed at this melt track overlap rate. As shown in Figure 12b, when the melt track overlap rate is 9.14%, from the P1 diagram, the surface powder in the overlapping zone has a good melting degree. However, from the P2 and P3 cross-sections, due to insufficient melting depth in the overlapping zone, the powder below the overlapping area cannot be melted well. From the red contour line in the P2 cross-section, it can zone be seen that there are many pores remaining between unmelted powders below the overlapping zone. As the melt track overlap rate continues to increase, when the melt track overlap rates are 14.3%, 20%, 25.7%, and 31.4%, respectively, from the P3 cross-sectional diagrams of Figure 12c–f, it can be seen that the melting width and depth of the overlapping zone continue to increase. From the red contour line in the P2 cross-sectional diagram, it can also be seen that at these melt track overlap rates, the powder at the bottom of the overlapping zone can be completely melted, and basically there are no obvious pores.

#### 4.3.2. Analysis of the Welding State Between the Upper and Lower Melt Tracks

**Figure 13:**
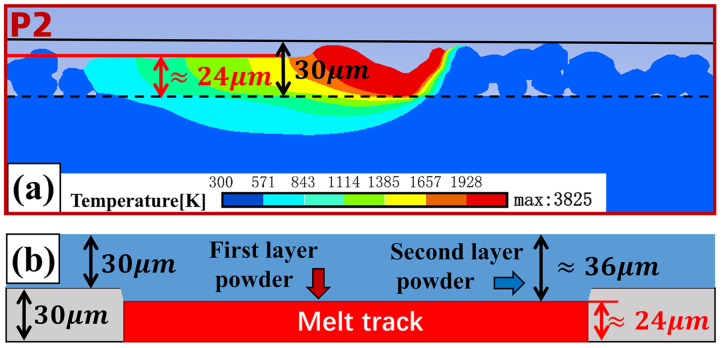
Increase in the thickness of the upper layer powder after the lower layer powder melts: (**a**) melt track simulation diagram, and (**b**) schematic diagram of powder layer thickness.

As can be seen from P2 in Figure 12c, when the melt track overlap rate is 14.3%, the overlapping effect between adjacent melt tracks is good, and there are no pores generated by unmelted powder in the overlapping zone. However, as can be seen from P3 in Figure 12c, when the melt track overlap rate is 14.3%, the melting depth of the overlapping zone obtained by model measurement is approximately 30.4 µm, which is almost the same as the single-layer powder spreading thickness of the powder bed. In the research of He et al. [25], it was found that when the depth of the overlap zone of the first layer is exactly equal to the thickness of the powder bed, the welding between the upper and lower layers may be non-dense. However, they did not explain the specific reasons. In fact, in the actual forming process, the lower layer powder will have phenomena such as metal evaporation and densification during the melting process. Therefore, the actual thickness of the melt track formed after the lower layer powder melts and solidifies is less than 30 µm. When the second layer of powder is spread, the upper layer powder will cover the lower layer melt track, which will make the actual thickness of the upper layer powder exceed 30 µm. As shown in Figure 13a, through melt track simulation, it can be measured that when the powder spreading thickness is 30 µm, the thickness of the first-layer melt track formed after the lower layer powder melts is about 24 µm. Then, the increase in the thickness of the second-layer powder above the molten track should be about 6 µm, and the actual thickness of the second layer powder is about 36 µm, as shown in Figure 13b. Therefore, when the melt track overlap rate is 14.3%, the connection effect between the upper and lower melt tracks in the overlapping zone may not be good. It is necessary to simulate and analyze the welding effect of the upper and lower melt tracks at this melt track overlap rate.

**Figure 14:**
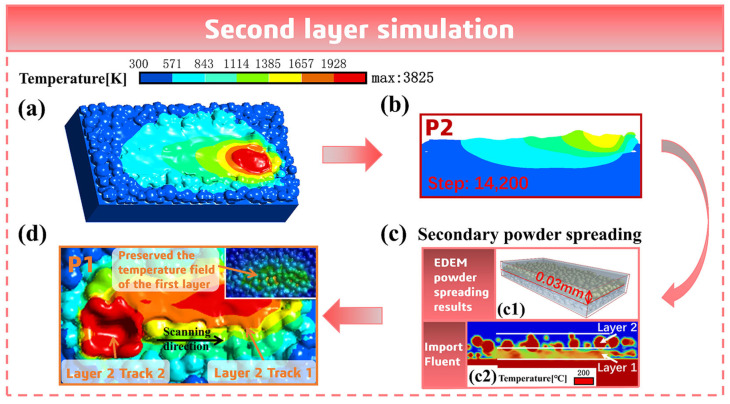
(**a**) The end state of the first layer simulation. (**b**) The residual temperature field after waiting for 600 time steps. (**c**) Laying the second layer of powder: (**c1**) the powder bed model after secondary powder spreading by EDEM 2022.2, and (**c2**) importing into Fluent 2023 R1 software. (**d**) Conducting the melt track simulation of the second layer.

This simulation is carried out on the basis of the simulation results of the first layer of double melt tracks. The state at the end of the first layer simulation is shown in Figure 14a. Considering the time difference existing in interlayer formation, it is set to wait for 600 time steps after the end of the first layer melt track simulation, which will cause the temperature on the surface of the first layer melt track to drop somewhat. The residual temperature field after waiting is shown in Figure 14b. Then, the EDEM 2022.2 software is used to spread the powder of the second layer on the basis of the double melt track model of the first layer and import it into the Fluent 2023 R1 software, as shown in Figure 14(c1,c2). Subsequently, under the condition of preserving the temperature field of the first layer melt track, the simulation of the second layer is carried out, as shown in Figure 14d.

**Figure 15:**
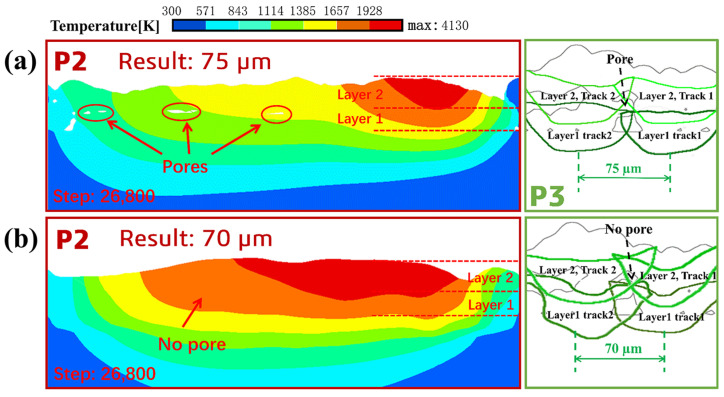
Simulation results of the second layer melt track: (**a**) melt track overlap rate is 14.3%, and (**b**) melt track overlap rate is 20%.

Figure 15a shows the cross-sectional views of P2 and P3 after the end of the second layer simulation when the melt track overlap rate is 14.3%. The dark green contour line is the contour of the first layer melt track, and the light green contour line is the contour of the second layer melt track. From the simulation results, it can be seen that due to the retention of the temperature field of the first layer, the temperature field of the second layer melt track will have an “overlay” effect on the temperature field of the first layer melt track, resulting in heat accumulation, leading to an increase in the width and depth of the second layer melt track. However, the melting depth in the overlapping zone of the second layer melt track is still not sufficient to completely melt the powder layer with a thickness of about 36 µm. This will cause a small amount of unmelted powder and pores between the powder grains to exist below the overlapping zone of the second layer, as shown in P2 and P3 of Figure 15a.

The second layer melt track is simulated in the same way when the melt track overlap rate is 20%, as shown in Figure 15b. From the simulation results, it can be seen that the pores existing in the cross-section of P2 have completely disappeared. From P3, it can be seen that the melting depth in the overlapping area has exceeded the thickness of the 36 µm powder layer, and welding between the upper and lower melt tracks has been achieved. Therefore, according to the simulation results, it can be explained that the workpiece formed when the melt track overlap rate is 20% has better density and connection performance between the upper and lower melt tracks compared to the workpiece formed when the melt track overlap rate is 14.3%.

### 4.4. Temperature Field Changes in the Overlapping Zone

**Figure 16:**
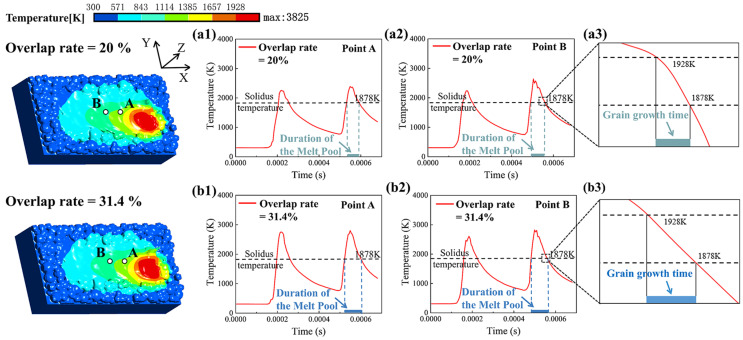
When the melt track overlap rate is 20%: (**a1**) temperature change curve at point A, (**a2**) temperature change curve at point B, and (**a3**) solidification segment curve at point B. When the melt track overlap rate is 31.4%: (**b1**) temperature change curve at point A, (**b2**) temperature change curve at point B, and (**b3**) solidification segment curve at point B.

As can be seen from the above simulation results, when the melt track overlap rate is 25.7% and 31.4%, the zone of the overlapping region between melt tracks is relatively large, and all the powder in the overlapping zone can be well melted. However, it can also be seen from the experimental results that an overly large overlapping zone will also have a certain negative impact on the mechanical properties of the workpiece after forming. Lo et al. [23] also proposed that the distance between the melting tracks is the main factor affecting the internal temperature change in the overlap zone. Moreover, the slow heat dissipation in the overlap area will affect the growth of internal micro-grains, thereby influencing the mechanical properties of the parts. In the study of Lo et al. [23], there is relatively little discussion of the impact of heat accumulation in the overlap zone. Therefore, through the simulation model, the influence of the melt track overlap rate on the temperature field of the overlapping zone under the condition of a high overlap rate (20% and above) is further studied. Taking the melt track overlap rates of 20% and 31.4% as the objects, in the simulation model, two points at the center of the overlapping zone of the double melt track model are extracted, respectively, namely point A (0.35, 0, −0.025) and point B (0.3, 0, −0.025). The temperature change curves at these points are shown in Figure 16(a1,a2,b1,b2). As can be seen from the figure, the maximum temperature inside the overlapping zone that undergoes the second melting is about 200 K higher than that of the first melting. This is caused by the residual heat of the first melt track leading to heat accumulation, and the molten pool in the overlapping zone after the second melting exists for a longer time than that after the first melting. Then, by extracting the solidification segment curve of molten pool metal with a temperature between 1878 K (solidus temperature of Ti-6Al-4V) and 1928 K (liquidus temperature of Ti-6Al-4V), as shown in Figure 16(a3,b3), it can be found that when the melt track overlap rate is 31.4%, the time available for grain growth in the overlapping zone is relatively longer. The reason is that the heat accumulation due to the increase in the overlapping zone will not only lead to a higher peak temperature of the molten pool but also make it more difficult for the overlapping zone to dissipate heat. This also means that the β-Ti grains in the overlapping zone will have more time to become coarser and thicker, which is also relatively consistent with the experimental results.

## 5. Conclusions

In this paper, by combining simulation and experiment, the influence of different melt track overlap rates on pores and temperature fields in the overlapping zone in the SLM forming process at macro and micro scales is studied. The forming quality of samples at different melt track overlap rates is analyzed and discussed. The main conclusions are as follows:

(1) SLM forming experiments were conducted on Ti-6Al-4V alloy. The experimental results show that when the melt track overlap rate is 20%, the density of the sample is 98.73%. Without special treatment to eliminate the residual stress of the samples, the tensile strength is 1092.95 ± 11.05 MPa, and the grains in the overlapping zone do not grow significantly. Compared with other samples, the overall mechanical properties of this sample are better;
(2) Using Fluent 2023 R1 software, a double melt track model of the SLM forming process was established. The double melt track forming process of Ti-6Al-4V alloy at six different melt track overlap rates (0%, 9.14%, 14.3%, 20%, 25.7%, 31.4%) was simulated. According to the cross-sectional diagram of the double melt tracks, the relationship between the melt track overlap rate and the residual pores was analyzed. The results show that when the melt track overlap rate is 0%, there will be a large number of pores in the overlapping zone caused by unmelted powder. When the melt track overlap rate is 9.14%, although the surface forming quality is intact, due to the smaller depth of the overlapping zone, the powder at the bottom of the overlapping zone cannot be fully melted. Therefore, there are also pores at the bottom of the overlapping zone;
(3) Through the simulation of the second layer melt track, the forming effect of the double-layer double melt track was further studied. The results show that in the actual forming process of SLM, due to the melting and densification effect of the lower powder layer, the actual powder spreading thickness of the second layer is slightly greater than 30 µm (about 36 µm). Therefore, when the melt track overlap rate is 14.3%, the melting depth in the overlapping zone of the second layer is still not enough to ensure the welding between the upper and lower melt tracks. When the melt track overlap ratio is 20%, the welding between the upper and lower melt tracks is better, and there are fewer pores in the sample;
(4) The influence of different overlap rates on the temperature field in the overlapping zone was studied through simulation. The simulation results show that an excessive melt track overlap rate will increase the peak temperature of the molten pool in the overlapping zone, and result in slow heat dissipation in the overlapping zone. The solidification segment curve shows that excessive remelting of materials in the overlapping zone will also prolong the time of grain growth in the overlapping zone;
(5) According to the simulation and experimental results, a 20% melt track overlap rate leads to a better welding state between the upper and lower melt tracks, fewer pores in the overlapping zone, and no excessive grain growth. Therefore, the sample with a 20% melt track overlap rate achieves the highest tensile strength. The experimental results show a good degree of agreement with the simulation results.

In future research, it is advisable to consider how to supplement and calculate the residual thermal stress data in this model and export it completely for further mechanical calculation and analysis. Additionally, based on this model, the influence of different rotation angles between layers on the quality of formed samples can be discussed by changing the rotation angle between layers during the forming process.

## Figures and Tables

**Table 1:** Chemical composition of Ti-6Al-4V powder used in the selective laser melting (SLM) process [10,11].

Ti-6Al-4V	Ti	Al	V	Fe	Y	C	O	N	H	Other
Wt.%	89.59	6.13	3.95	0.12	<0.005	0.006	0.065	0.012	0.005	0.12

**Table 2:** Process parameters of the simulation process of SLM.

Parameters	Values
Laser radius	35 µm
Laser power	240 W
Scanning speed	1200 mm/s
Powder thickness	30 µm
Scanning direction	Left to right

**Table 3:** SLM Process parameter sets for samples No. 1 to No. 12.

Samples	Scanning Distance (µm)	Overlap Rate (%)
1, 7	60	31.4
2, 8	65	25.7
3, 9	70	20.0
4, 10	75	14.3
5, 11	80	9.14
6, 12	100	0

**Table 4:** Thermophysical properties of Ti-6Al-4V in the SLM process [38].

Parameters	Values and Units
Density (solid)	4420 kg/m^3^
Density (liquid)	3920 kg/m^3^
Surface tension	1.525 N/m
Specific heat capacity (solid)	412.7 J/(kg·K)
Specific heat capacity (liquid)	831 J/(kg·K)
Radiation coefficient	1
Liquidus temperature	1928 K
Solidus temperature	1878 K
Boiling point	3315 K
Thermal conductivity (solid state)	7.955 W/(m·K)
Thermal conductivity (liquid state)	12.752 W/(m·K)
Latent heat of fusion	286 kJ/kg
Latent heat of vaporization	9700 kJ/kg
Coefficient of thermal expansion (CTE)	8.0 × 10^−6^ (K^−1^)
Molar mass	46.49 g/mol
Laser absorption rate	60%

## Data Availability

The original contributions presented in this study are included in the article. Further inquiries can be directed to the corresponding author.
